# Generation of Processed‐to‐Raw Food Conversion Factors for Estimating Food Raw Material Intake From Various Processed Foods: Valuable Tools for Dietary Exposure Assessments

**DOI:** 10.1002/fsn3.70064

**Published:** 2025-06-01

**Authors:** Jiyun Baek, Yerim Han, Chaehyun Kim, You Rim Kang, Seung Hui Baik, Yoon Jung Park, Ji‐Myung Kim, Youngjoo Kwon

**Affiliations:** ^1^ Department of Food Science and Biotechnology Ewha Womans University Seoul Republic of Korea; ^2^ Department of Nutritional Science and Food Management Ewha Womans University Seoul Republic of Korea; ^3^ Graduate Program in System Health Science and Engineering Ewha Womans University Seoul Republic of Korea; ^4^ Department of Food and Nutrition Shinhan University Uijeongbu Gyeonggi Republic of Korea

**Keywords:** dietary exposure assessment, migration rate, partition ratio, percentage yield, processed foods, processed‐to‐raw food conversion factor

## Abstract

Estimating food intake is an important means of assessing dietary exposure to chemicals. However, while sources of concentration data (e.g., safety levels and nutrient content) are often available for food raw materials, foods are consumed in both processed and raw forms. Therefore, processed food intake levels must be properly converted to those of their constituent ingredients to accurately estimate food intake. On this premise, the current study aimed to generate processed‐to‐raw food conversion factors (PRCFs). To generate PRCFs, two approaches were primarily employed. One approach involved the percentage yield method, wherein conversion factors were generated by calculating reverse percentage yield. For foods that had undergone simple processing procedures (e.g., soaking and dehydration) as a whole foods, percentage yield was exclusively used. Nevertheless, numerous processed foods (e.g., milled grains and butter) are simultaneously produced from distinct fractions after undergoing separation from their initial raw materials. For these foods, PRCFs were derived using partition ratios in combination with reverse percentage yield. For the remaining processed foods (e.g., vinegars and tea infusions) in which weight changes were not easily traceable, the migration rate method, which calculates the content of specific components in the final processed food relative to that in the initial food ingredients, was utilized. The literature was extensively reviewed to collect the required data. In addition, polyphenol content was directly measured using the Folin–Ciocalteu assay to derive polyphenol migration rates for tea infusions and stocks prepared with spices. In total, the current study generated 120 PRCFs across diverse processing procedures and food types. These factors will serve as a valuable tool for the accurate estimation of food intake, thereby facilitating adequate dietary exposure assessments associated with food chemicals, such as pesticide residues, food contaminants, nutrients, and other substances.

## Graphical abstract

One approach of generating processed‐to‐raw food conversion factor was the percentage yield method wherein the weight ratio of initial raw materials to final processed products was calculated. For foods that had been processed as a whole food, percentage yield was exclusively used, whereas partition ratios were also used for foods that had been produced after separation from their initial raw materials. The other approach entailed the migration rate method, which calculated the content ratio of specific components in the final processed foods relative to their initial raw ingredients.

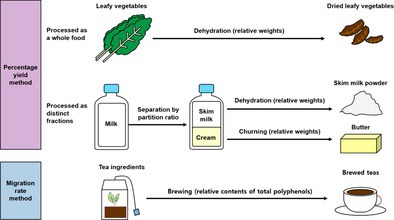

## 1 Introduction

Dietary exposure assessment (intake assessment) is the process of estimating the quantity of a food chemical ingested through food. It is an important means of monitoring nutritional status and dietary exposure levels to hazardous chemicals (e.g., pesticide residues and food contaminants) in the population. Foods are consumed both as raw ingredients and processed products. However, safety standards for numerous hazardous chemicals have been established at the food raw material level. In addition, information regarding nutrient content is predominantly available for food ingredients. Therefore, for precise estimation, processed food intake levels have to be adequately converted to those of their constituent food ingredients.

Therefore, conversion factors have been applied to estimate food intake (Kweon 2012). Nonetheless, these factors are based on calculations that solely account for changes in sold contents relative to those in food weights for processed foods previously subjected to drying, heating, or freezing, where water is either added or removed. Different processing techniques result in discrete changes in food ingredients. Therefore, developing methods of generating conversion factors that consider such differences across various processing procedures is requisite to accurately assessing food intake at the raw material level. Furthermore, even with the same procedure, the same conversion value may not be usable across diverse food ingredients, as each food ingredient exhibits distinct physicochemical properties (EFSA 2015; EFSA et al. 2019; Schmid et al. 2020). In this regard, the European Food Safety Authority (EFSA) utilized reverse yields as well as recipes from different sources to estimate the amounts of raw primary commodities in each reported processed food consumed, thereby successfully transforming the Comprehensive European Food Consumption Database into raw primary commodity (RPC) consumption data (EFSA 2015; EFSA et al. 2019). This RPC consumption database enables the dietary exposure assessment of chemicals, particularly when RPC occurrence data are predominantly available (EFSA 2015; EFSA et al. 2019), suggesting the importance of conversion factors as a tool in the exposure assessment of food chemicals. However, most of the reference data used to develop these factors were generated before 2002. Furthermore, certain food processing procedures (e.g., oil production) involve separation. Reverse yield factors utilized by the EFSA do not consider food partitioning, potentially leading to an overestimate of RPC consumption, as it is assumed that RPCs are consumed in their entirety. Additionally, reverse yields may not be used to estimate the quantity of consumed raw materials on certain occasions, as in the cases of assorted teas and broths/stocks, where only the brewed or extracted portion is consumed.

Therefore, the current study aimed to generate processed‐to‐raw food conversion factors (PRCFs) that translate the quantities of particular processed foods into those of their respective food ingredients for each food type and processing (or preparation) procedure in an effort to develop accurate food intake assessment methods. In this study, two approaches were primarily employed to derive PRCFs: (1) the percentage yield method was used to evaluate most processed foods, and (2) the migration rates of specific compounds during processing were estimated for processing procedures involving substantial alterations to food ingredients (e.g., extraction and brewing). An extensive literature search was conducted to collect information on percentage yields and migration rates across various food types and processing conditions. To minimize the effects of variability in processing conditions (e.g., temperature and time) across different studies, the collected values were averaged for each food type under each processing procedure. This approach also facilitated the extrapolation of PRCFs from those of similar food types, addressing data gaps for foods with limited information. All conversion factors developed in the present study are based on the most available information and are intended to reflect current conditions and practices. Moreover, polyphenol content was directly measured using the Folin–Ciocalteu assay to yield polyphenol migration rates, as they could not be acquired from the literature. The PRCFs generated in the current study will serve as a valuable tool for the accurate estimation of food intake, thereby facilitating adequate exposure assessment associated with food chemicals.

## 2 Materials and Methods

### 2.1 Study Design

**FIGURE 1:**
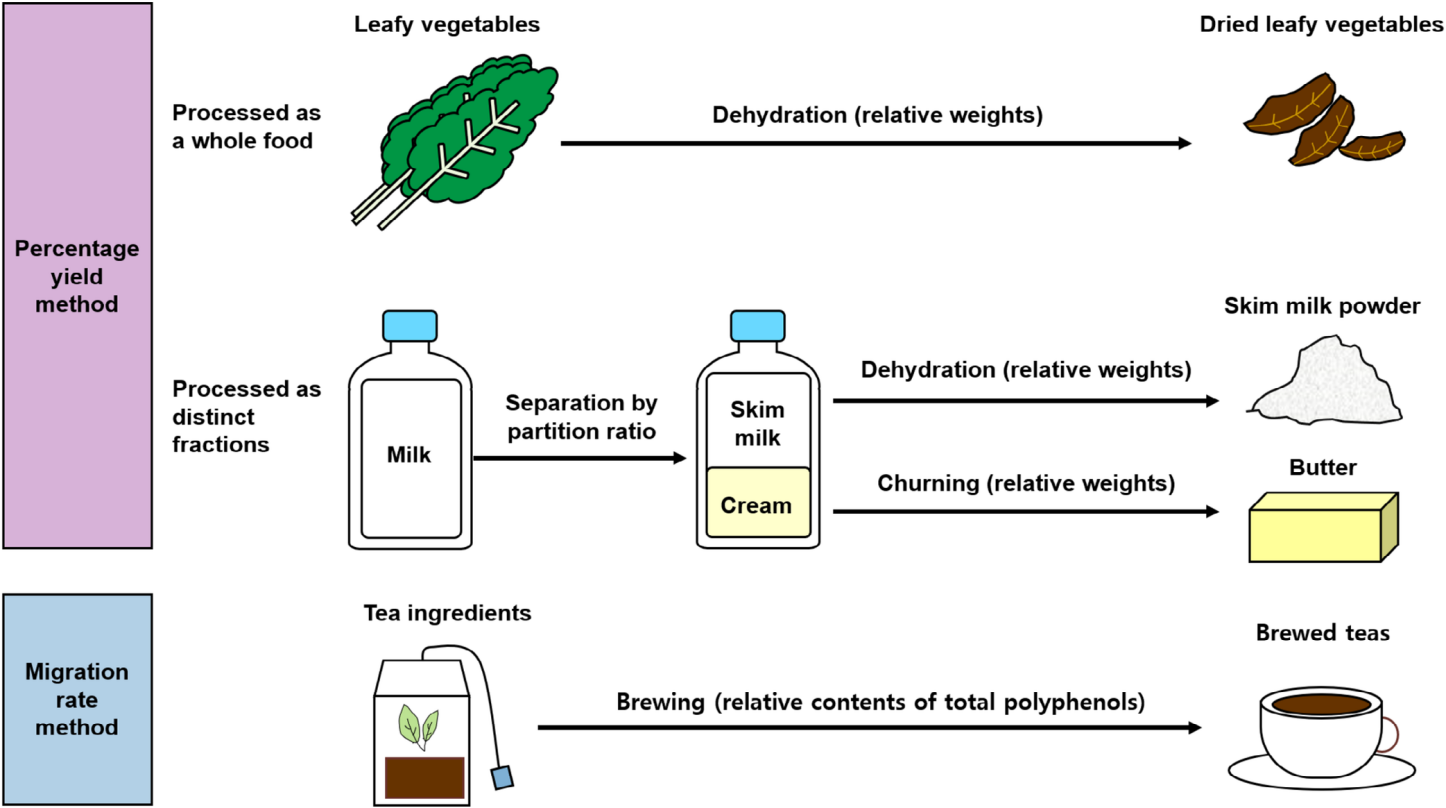
Generation of processed‐to‐raw food conversion factors primarily using two methods. One approach was the percentage yield method wherein the weight ratio of initial raw materials to final processed products was calculated. For foods that had undergone simple processing procedures as a whole food, percentage yield was exclusively used. For foods that had been produced from distinct fractions after undergoing separation from their initial raw materials, conversion factors were derived using partition ratios in combination with percentage yield. The other approach entailed the migration rate method, which involved calculating the content ratio of specific components in the final processed foods relative to their initial raw ingredients.

Figure 1 depicts the process of generating PRCFs using two approaches in the current study. In generating these conversion factors, major processed foods were targeted based on the food processing techniques described in the Food Classification and Description System for Exposure Assessment developed by the EFSA and a database of the bisphenol A content of foods commonly consumed in Korea (Lee et al. 2024). Targeted processed foods are produced via various procedures, ranging from grinding grain into flour to home cooking to complicated industrial processing procedures, including soy sauce production and alcoholic beverage production.

Reverse percentage yields (weight changes of food ingredients during processing) were used to generate conversion factors (percentage yield method) for most processed foods. Nevertheless, in cases where food ingredients undergo substantial alterations during processing (e.g., vinegar production), the migration rates of specific components from initial food ingredients into final processed foods were utilized (migration rate method). The migration rate method was also applied to food ingredients not directly consumed but rather extracted into cooking liquids (e.g., broths/stocks and teas) for consumption. The selection criteria for specific components were based on their abundance and presence in both food raw materials and their resulting products, thus being easily traceable. Meanwhile, during processing, the forms of these components should not be substantially modified via denaturation or degradation. The obtained selected components comprised α‐acids, total polyphenols, total solids, and carbohydrates for beer, herbal infusions, broths/stocks, and alcoholic beverages and vinegars, respectively (Blanco et al. 2006; Jun et al. 2021; Moon and Cheong 2018; Na et al. 2013; Pasrija and Anandharamakrishnan 2015).

The percentage yields and migration rates of targeted processed foods were acquired via a comprehensive literature search and from available databases (Section 2.2); this excludes the calculation of migration rates for herbs and spices during brewing and simmering. The total polyphenol content was directly measured from herbs or spices and their infusions or prepared stocks to generate conversion factors for brewed teas and stocks prepared with spices (Section 2.3). Upon PRCF formulation within respective processing procedure categories and food type combinations, values were averaged to generate a conversion factor for the corresponding processed food type to reflect inter‐study variation arising from the use of varying conditions (time and temperature).

### 2.2 Literature Review

To determine the ratio of the weight of the finished product to that of the initial raw material, studies evaluating weight changes during processing were sought using search engines, such as Google Scholar, PubMed, and EBSCOhost. Keywords encompassing “yield,” “percentage yield,” and “production yield” along with specific processed foods, such as “soy sauce,” “powdered egg,” and “gouda cheese” were used. Resultantly, 140 scientific articles published in 1984–2024 were retrieved. In addition, manufacturers' websites were referenced to obtain information on weight changes during the production of dairy products, bean curds, soybean paste, and starch. Percentage yields were also calculated using data from two sources: (1) the database for processed food weights (KHIDI 2007) and (2) the nutritional composition of processed foods (MFDS 2020). These databases included information on the weight changes of diverse food ingredients during typical household cooking procedures.

Similarly, to generate conversion factors using the migration rate method, the content of specific components in the initial food ingredients and final processed foods was collected via literature review. In total, 47 scientific articles published in 2000–2024 were retrieved. In addition, food composition data from FoodData Central (USDA 2022) and the Korean Food Composition Database (NAS 2023) were used to obtain the concentrations of components in diverse foods. The total solid content of broth/stock was also secured from food nutrition labels provided by manufacturers.

### 2.3 Measurement of the Polyphenol Content of Teas, Stocks, and Their Raw Materials

Polyphenols are abundant in both raw and infused teas (or stocks) owing to their water‐soluble nature (Fecka and Turek 2007). Furthermore, their structures are relatively stable under brewing or simmering conditions (Fecka and Turek 2007; Pasrija and Anandharamakrishnan 2015). Therefore, the total polyphenol content was measured to estimate the quantities of teas, herbs, and spices that had migrated from raw materials to their prepared teas or stocks. Teas were classified into five categories based on the plant parts from which they had been extracted (e.g., leaves and roots), as extraction efficiency is potentially influenced by them (Ghadage et al. 2017; Karabegovic et al. 2014). Tea types popularly consumed worldwide were selected for each category (plant parts), as shown in Table 1. Single‐ingredient tea products were exclusively used to ensure that the polyphenols present in the brewed forms had migrated from a specific raw material. Likewise, spices widely used to prepare stocks were selected for each category (Table 1). In total, 27 tea products and 12 spices were purchased online (SSG.COM Inc., Seoul, Korea, http://www.ssg.com) between September 2023 and February 2024. They were in favorable condition—with > 1 year remaining until their best‐before date, as indicated on their packaging—and kept unopened until analysis.

**TABLE 1:** Teas, herbs, and spices used to determine the migration rates of total polyphenols.

Plant parts	Tea/herb/spice	Brand
Leaf	Black tea ( *Camellia sinensis* )	Lipton (United Kingdom)
		TWG (Singapore)
		Twinings (United Kingdom)
	Green tea ( *Camellia sinensis* )	Ahmad tea (United Kingdom)
		Lipton (United Kingdom)
		Osulloc (Korea)
	Peppermint (*Mentha piperita* L.)	Lipton (United Kingdom)
		Teazen (Korea)
		Twinings (United Kingdom)
	Rooibos ( *Aspalathus linearis* )	Clippers (United Kingdom)
		Lipton (United Kingdom)
	Bay leaf ( *Laurus nobilis* )	Mokhwa (Korea)
Flower	Chamomile ( *Matricaria recutita* L.)	Lipton (United Kingdom)
		TWG (Singapore)
	Hibiscus ( *Hibiscus sabdariffa* L.)	Royal orchard (Korea)
		Teazen (Korea)
Root	Burdock ( *Arctium lappa* L.), roasted	Ssanggye (Korea)
		Teazen (Korea)
	Solomon's seal (*Polygonatum odoratum var. Pluriflorum* (Miq.) Ohwi), roasted	Dongsuh (Korea)
		Teazen (Korea)
Fruit/seed	Barley (*Hordeum vulgare var. Hexastichon* (L.) Aschers.), roasted	Damtuh (Korea)
		Danongwon (Korea)
		Dongsuh (Korea)
	Cassia seed ( *Cassia tora* L.), roasted	Damtuh (Korea)
		Dongsuh (Korea)
		Ssanggye (Korea)
	Rosehip ( *Rosa canina* L.)	Epanie (Korea)
		Royal orchard (Korea)
Bark	Cinnamon ( *Cinnamomum verum* )	Tojongherb (Korea)
		Oherb (Korea)
		Pureundeulpan (Korea)
Flower bud	Clove ( *Syzygium aromaticum* )	Naturefood (Korea)
Fruit/seed (hard)	Black pepper ( *Piper nigrum* )	CostcoKorea (Korea)
	Coriander ( *Coriandrum sativum* )	Shinyoungspice (Korea)
	Nutmeg ( *Myristica fragrans* )	Cannamela (Italia)
	Star anise ( *Illicium verum* )	Hanbangmaster (Korea)
Fruit/seed (soft)	Caraway ( *Carum carvi* L.)	Shinyoungspice (Korea)
	Cumin ( *Cuminum cyminum* )	Naturefood (Korea)
	Fennel ( *Foeniculum vulgare* )	Shinyoungspice (Korea)

Purchased teas and spices were extracted for total polyphenol measurements using the International Organization for Standardization (ISO) 14502‐1:2005 protocol (ISO 2005), with slight modification. Briefly, 0.2 g of each raw material was extracted using 5 mL of 70% methanol in a 70°C water bath for 1 h. During incubation, the mixture was occasionally vortexed to facilitate extraction. Afterward, the extract was cooled to room temperature and centrifuged at 3500 rpm for 10 min. The resulting supernatant was collected and extraction was repeated for the remaining residues using another 5 mL of 70% methanol. Supernatants from three extractions were pooled and adjusted to a final volume of 15 mL. The obtained polyphenol extracts were diluted with water before determining the total polyphenol content. Tea infusions were prepared according to the respective manufacturers' instructions written on the corresponding tea packages. To reproduce stock preparation, 1 g of each spice was placed in 200 mL of water and the suspension was heated to boiling point for 2 h on a heating plate. In a preliminary study, no noticeable differences in extraction efficiency according to simmering time occurred within 1–6 h.

The total polyphenol content of the extracts was determined using the Folin–Ciocalteu colorimetric assay. Polyphenol extracts from the raw materials of teas or spices and their brewed or simmered versions (100 μL) were mixed with 0.5 mL of 10% Folin–Ciocalteu solution (Sigma‐Aldrich, St. Louis, MO, USA) and subsequently with 0.4 mL of 7.5% sodium carbonate solution. Following 1‐h incubation, the absorbance values of the mixtures were measured at 765 nm using a VersaMax Microplate Reader (Molecular Devices, Sunnyvale, CA, USA). All experiments were conducted in triplicate. Gallic acid (Sigma‐Aldrich, St. Louis, MO, USA) was used as a standard. The results are expressed in gallic acid equivalents per gram of tested sample, and the total polyphenol content of the raw tea materials was compared with that of reported values determined using the Folin–Ciocalteu assay in previous studies. Determined polyphenol contents were used to calculate total polyphenol migration rates (Section 2.4.2).

### 2.4 Generation of Conversion Factors

#### 2.4.1 Percentage Yield Method

The percentage yield of processed foods is considerably influenced by the processing technique employed and the physicochemical properties of the raw materials undergoing processing. Therefore, within each processing procedure, PRCFs were separately generated according to the physicochemical nature of each specific food type.

Using retrieved information on weight changes during various food processing procedures, reverse percentage yields were calculated, as described in Equation (1). Here, parts removed prior to processing (inedible portions) were not included in the initial weights of the food ingredients. This method was applied to relatively simple food processing procedures that chiefly involve heating, freezing, soaking, and dehydration. However, numerous processed foods are simultaneously produced from individual compartments after undergoing separation from their initial raw materials; each fraction yields a discrete product. Under such circumstances, the conversion of a product's weight into that of its initial ingredients exclusively using reverse percentage yield eventually causes the overestimation of the initial raw material weight owing to the summation of converted weights (weights equivalent to those of the raw ingredients) from multiple products. For example, fresh eggs comprise 67% egg white and 33% egg yolk (USDA 1992). On estimating raw material intake from products, each egg yolk and egg white weight is converted back to egg's corresponding initial weight by factors of reversing percentage yield (1.49 and 3.03 for egg yolk and egg white, respectively). Therefore, the combined consumption of 0.67 g of egg white and 0.33 g of egg yolk results in a total consumption of 2 g instead of 1 g of egg, as each would have been converted to approximately 1 g. Therefore, partition ratios were used to correct initial weights owing to fractionation. In other words, only the egg white portion (by weight) rather than the entire egg is expected to yield egg white; thus, to derive PRCFs, a partition ratio of 0.67 was considered in addition to the ratio of the initial weight (egg) to the final product weight (egg white). Consequently, for processed foods produced from separated parts of the initial raw ingredient, PRCFs were generated using Equation (2).

(1) $$\text{Percentage yield}=\frac{\text{Weight of initial food ingredient}}{\text{Weight of finished processed food}}$$

(2) $$\text{Percentage yield}=\frac{\text{Weight of initial food ingredient}\times \text{partition ratio}}{\text{Weight of finished processed food}}$$

The separation of egg white and egg yolk from whole eggs principally involves physical separation without substantial alterations in chemical composition (e.g., increase or decrease in water content). In fact, partition ratios are equal to the yield ratios of the final products produced from individual raw material fractions, resulting in a conversion factor of “1” owing to calculations entailing the multiplication of partition ratios using reverse percentage yields. Simply put, percentage yields calculated based on the weights of separated materials approximate 1. Likewise, milling physically separates whole grains into brans, germs, and refined grains at mere ratios of 8%–15%, 2%–6%, and 82%–90%, respectively (Ndolo and Beta 2013; Van Hoed et al. 2006); thus, they have equal partition ratios, similar to the ratios of the final products derived from whole grains. Vegetable oil produced via press extraction also entails the separation of oils and oil cakes during processing. Most vegetable oils are also produced via solvent extraction, wherein additional oils are extracted from oil cake to increase oil yield (Bhuiya et al. 2020). Solvent extraction involves solvent addition; nonetheless, the added solvents are completely removed during production. Therefore, partition ratios were presumably similar to oil and oil cake production ratios, as in vegetable oil production via mechanical pressing. Similarly, juicing fruits and vegetables physically separates them into juice and pulp portions. Therefore, a conversion factor of “1” was uniformly assigned to processed foods derived via polishing, milling, vegetable oil production, and fruit and vegetable juicing. However, if post‐separation downstream procedures modify food composition (e.g., water content), weight change will be reflected in percentage yield. When processing procedures initially included pre‐separation hydration steps, as in soybean processing into final products (e.g., tofu, soy milk, soy sauce, soybean paste, etc.), partition ratios were based on the initial combined weights of water and raw materials.

#### 2.4.2 Migration Rate Method

The percentage yield method may not be appropriate in cases wherein processing procedures elicit substantial changes in food ingredients, as in vinegar and alcohol production, rendering it difficult to follow weight changes. Similarly, foods can be consumed as extracts infused into cooking liquid or water, as in the case of broth or stock prepared from various plants or meats as well as hop or tea infusion. Therefore, the migration rates of specific components were employed where percentage yields were inapplicable. The selected components were α‐acids, total polyphenols, total solids, and carbohydrates for beer, herbal infusions, broths/stocks, and alcoholic beverages and vinegars, respectively. Their structures are relatively stable during processing, and they are easily traceable (Blanco et al. 2006; Jun et al. 2021; Moon and Cheong 2018; Na et al. 2013; Pasrija and Anandharamakrishnan 2015).

During alcoholic beverage and vinegar production, the carbohydrates contained in food ingredients are initially hydrolyzed to fermentable sugars and subsequently fermented into alcohol or acetic acid (Na et al. 2013; Park et al. 2010). All carbohydrates in raw materials presumably undergo complete breakdown into fermentable sugars, which are eventually converted into alcohol or acetic acid. Therefore, for vinegars and alcoholic beverages, migration rates were calculated using the carbohydrate content of raw materials and acetic acid or alcohol content of finished processed foods. As described in the percentage yield method (Section 2.4.1), the edible portion was exclusively considered when calculating migration rates, and the resulting values were averaged for each specific processing technique and food type. As such, for alcoholic beverages, conversion factors were independently generated based on the alcohol preparation method (alcoholic beverage type) and raw material similarity (carbohydrate type).

Migration rates were calculated as the ratio of a specific component's weight per 100 g of processed food to its weight per 100 g of the edible portion of the initial food ingredient (Equation (3)). To generate migration rates for alcoholic beverages and vinegars, Equations (4) and (5) were used, respectively. Here, theoretical fermentation yields of 0.51 and 0.67 were used for alcohol and acetic acid, respectively (Aiba et al. 1968; Sugaya et al. 1986).

(3) $$\text{Migration rate}=\frac{\text{Weight of component}\;\mathrm{per}\;100\;g\;\text{finished processed food}}{\text{Weight of component}\;\mathrm{per}\;100\;g\;\text{edible portion of initial food ingredient}}$$

(4) $$\text{Migration rate}=\frac{\text{Alcohol}\;\mathrm{by}\;\text{volume of produced alcoholic beverage}}{\text{Weight of carbohydrate in edible portion of initial food ingredient}\times 0.51}$$

(5) $$M\text{igration rate}=\;\frac{\text{Acetic acid}\;\mathrm{by}\;\text{volume of produced vinegar}}{\text{Weight of carbohydrate in edible portion of initial food ingredient}\times 0.67}$$

## 3 Results

### 3.1 PRCFs Generated Using the Percentage Yield Method

**FIGURE 2:**
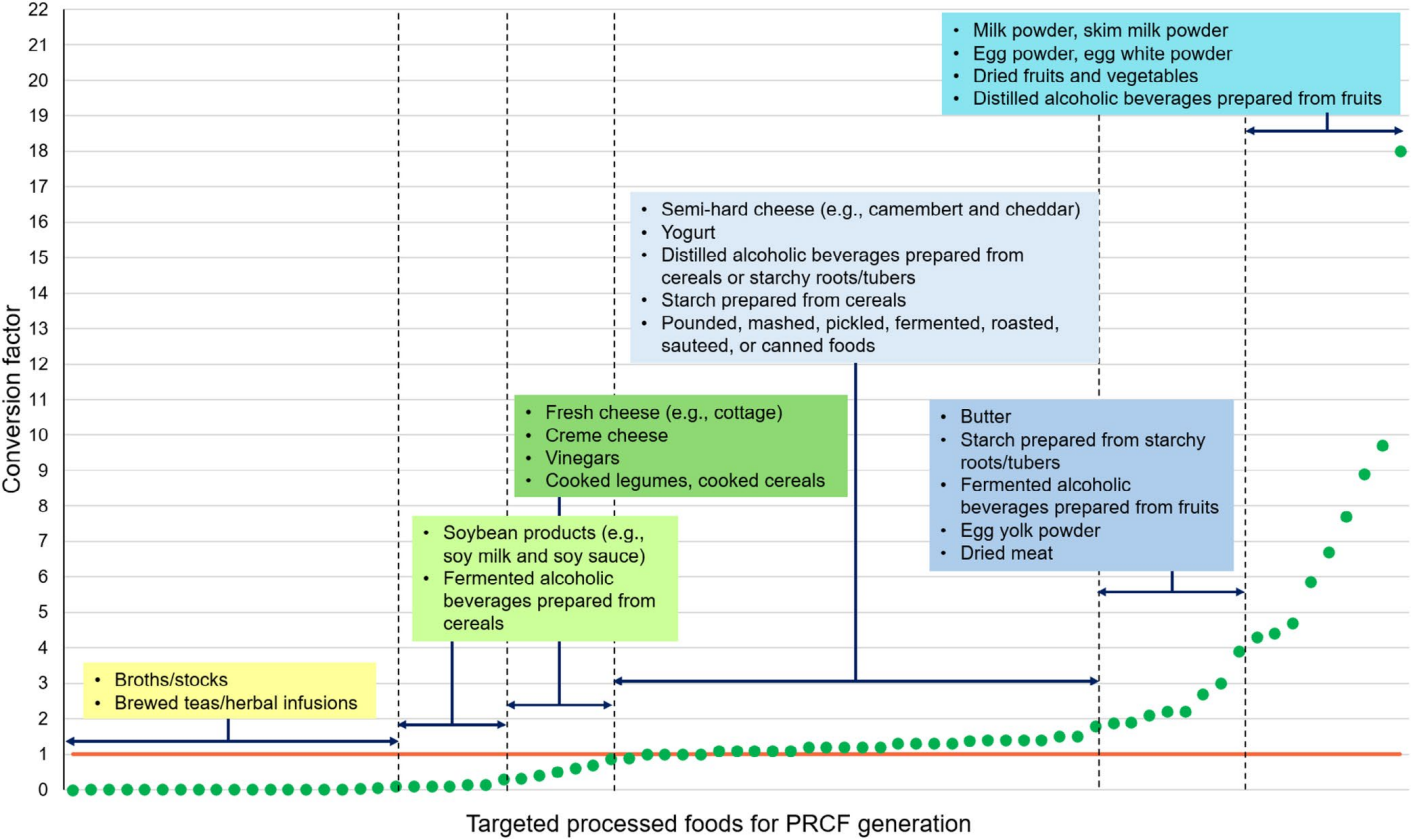
Processed‐to‐raw food conversion factors (PRCFs) generated for diverse processed foods. The current study generated a total of 120 conversion factors, which were influenced by processing procedure and food type. The PRCFs for each processing procedure and food type are listed in ascending order and subdivided into six groups based on their values. Certain foods, including polished cereals, milled products, egg yolk/egg white, vegetable/fruit juices, and vegetable oils, are solely produced via physical separation. Therefore, a conversion factor of “1” was assigned to processed foods derived via polishing, milling, vegetable oil production, and fruit and vegetable juicing (data not shown).

Upon generating PRCFs, the percentage yield method, which calculates reverse percentage yield (the ratio of the final product's weight to that of the initial food ingredient) was predominantly used. In total, 82 PRCFs were generated using this method (Tables 2 and 3). Among these, 32 were exclusively generated using percentage yields, which accounted for processed foods that had undergone minimal changes in composition or relatively simple water addition or removal procedures, such as boiling, freezing, soaking, and drying (Table 2). Mashing, pounding, pickle preparation, and fermentation cause minimal weight change in food ingredients. Therefore, the conversion factors for processed foods produced using these procedures approximated 1 (Table 2 and Figure 2). Notably, dehydration substantially reduces the final product's weight relative to that of the initial raw material, dramatically increasing PRCF values (Table 2 and Figure 2). Nevertheless, the derived PRCFs varied; jerky yielded the lowest (2.2), whereas fruiting vegetables/mushrooms generated the highest (18). Those of other raw materials, such as vegetables, fruits, milk, and eggs, ranged from 4 to 7 (Table 2).

**TABLE 2:** Processed‐to‐raw conversion factors generated using the percentage yield method.

Processing procedure	Processed foods	Initial food ingredients	Conversion factor^a^	Number of samples	References
Grinding	Flour	Cereals	1.4 ± 0.29	20	(EFSA et al. 2019; FAO 2000; Scholz et al. 2022)
	Powdered legumes, nuts, and spices	Legumes, nuts, and spices	1.0 ± 0.10	26	(EFSA et al. 2019; FAO 2000; Scholz et al. 2022; USDA 1992; Weber 2008)
Mashing	Mashed food	Fruits and vegetables	1.0	9	(EFSA et al. 2019; FAO 2000; USDA 1992)
Pickling	Pickles	Fruits and vegetables	1.1 ± 0.23	16	(EFSA et al. 2019; Scholz et al. 2022)
Yogurt production	Yogurt	Milk	1.3	1	(FAO 2000)
Flaking	Cereal flakes	Cereals	1.5 ± 0.59	10	(EFSA et al. 2019; FAO 2000; Scholz et al. 2022; USDA 1992)
Puffing	Puffed cereals	Cereals	1.1 ± 0.05	3	(Bergström 1994; EFSA et al. 2019; FAO 2000)
Canning	Canned foods	Fruits and vegetables	1.1 ± 0.19	18	(EFSA et al. 2019; FAO 2000; Scholz et al. 2022)
		Meat and fish	1.3 ± 0.08	3	(EFSA et al. 2019; Fisher et al. 2000)
Condensation/evaporation	Condensed/evaporated milk	Milk	2.7 ± 0.25	2	(FAO 2000)
	Jam	Fruits and vegetables	1.4 ± 0.09	24	(Bognár 2002; EFSA et al. 2019)
	Paste	Fruits and vegetables	4.4 ± 0.54	2	(FAO 2000; Scholz et al. 2022)
	Puree	Fruits and vegetables	2.2 ± 1.56	2	(FAO 2000; Scholz et al. 2022)
Dehydration	Dried fruits	Fruits	5.0 ± 2.09	25	(Choi et al. 2017; EFSA et al. 2019; FAO 2000; Lee and Kim 2015; Mwamba et al. 2017; Rho et al. 2000; Roh et al. 2002; USDA 1992)
	Dried vegetables	Leafy vegetables and herbs	6.7 ± 2.34	41	(EFSA et al. 2019; FAO 2000; Hossain et al. 2010; Jerkovic et al. 2001; Park and Chin 2023; USDA 1992; Weber 2008)
		Root, tuber, and bulb vegetables	5.0 ± 2.29	22	(Chang and Kim 2011; Choi et al. 2011; EFSA et al. 2019; FAO 2000; Gonçalves et al. 2017; Hwang and Kim 2024; Kim et al. 2022; Scholz et al. 2022; Shin et al. 2009; USDA 1992)
		Fruiting vegetables and mushrooms	18 ± 5.95	7	(Choi et al. 2022, 2014; EFSA et al. 2019; FAO 2000; Weber 2008)
	Jerky	Meat	2.2 ± 0.16	6	(Choi et al. 2008a; FAO 2000; Han et al. 2008a; Ndife et al. 2021; Song et al. 2014; Sorapukdee et al. 2016)
	Milk powder	Milk	5.52 ± 1.63	2	(Brindha and Kalai Nila 2020; FAO 2000)

Whole egg powder	Egg	3.9 ± 0.25	3	(Daramola‐Oluwatuyi et al. 2021; Evanuarini and Susilo 2022; USDA 1992)
Frying	Fried foods	Vegetables	1.8 ± 0.23	12	(Bergström 1994; Bognár 2002; EFSA et al. 2019; Scholz et al. 2022)
		Meat	1.4 ± 0.16	2	(Bognár 2002)
Roasting/sautéing	Cured meat	Meat	1.2 ± 0.31	29	(Baer and Dilger 2014; Bhattacharyya et al. 2007; Boler et al. 2011; Carvalho et al. 2014; Cosenza et al. 2003; Damaziak et al. 2016; Desmond et al. 2002; Du and Sun 2005; FAO 2000; Hullberg and Lundstrom 2004; Jridi et al. 2015; Kuo and Chu 2003; Kyle et al. 2014; Lee et al. 2008a; Loetscher et al. 2015; Lowell et al. 2018, 2019; Mor‐Mur and Yuste 2003; Nunez de Gonzalez et al. 2008; Person et al. 2005; Razmaite et al. 2008; Schilling et al. 2003; Seman et al. 2013; Sima et al. 2013; Sivendiran et al. 2018; Tavárez et al. 2014; Vickery and Rogers 2002)
	Roasted foods	Cereals and legumes	1.1 ± 0.05	8	(EFSA et al. 2019; Scholz et al. 2022)
		Vegetables	1.3 ± 0.50	8	(Bergström 1994; Bognár 2002; EFSA et al. 2019; Scholz et al. 2022)
		Meat and fish	1.5 ± 0.24	8	(Bergström 1994; Bognár 2002; EFSA et al. 2019)
Boiling	Boiled foods	Cereals and legumes	0.5 ± 0.20	10	(Bognár 2002; EFSA et al. 2019)
		Fruits and vegetables	1.1 ± 0.14	38	(Bergström 1994; Bognár 2002; EFSA et al. 2019)
		Meats and eggs	1.2 ± 0.26	16	(Bergström 1994; Bognár 2002; EFSA et al. 2019)
		Fish, seafood and invertebrates	1.2 ± 0.12	11	(Bergström 1994; Bognár 2002; EFSA et al. 2019)
	Homestyle soups^b^ (consommé, bouillon, “guk”, and “tang”)	Vegetables, meats, fish and seafood, dairy, and eggs	1.3 ± 0.61	86	(Bergström 1994; Bognár 2002; EFSA et al. 2019; FSANZ 2023; KHIDI 2007; MFDS 2020; USDA 2022)
Stewing	Homestyle stews^c^ (chili con carne, goulash, hot pot, and “jjigae”)	Vegetables, meats, fish and seafood, dairy, and eggs	1.4 ± 0.38	91	(Bergström 1994; Bognár 2002; EFSA et al. 2019; FSANZ 2023; KHIDI 2007; MFDS 2020; USDA 2022)

^a^ Conversion factors were calculated as reverse percentage yields (weight of the initial food ingredient relative to that of the finished processed food). Percentage yields from previous studies were averaged to generate factors, and they are presented as the mean ± standard deviation.

^b^ Thin soup styles, such as consommé and bouillon, are cooked with a large amount of water.

^c^ Thick stew styles, such as chili con carne, are cooked with vast chunks of ingredients over a prolonged period.

**TABLE 3:** Processed‐to‐raw conversion factors generated for processed foods produced after separation from initial raw materials using partition ratios and percentage yields.

Processing procedure	Initial food ingredients	Compartment	Partition ratio^a^	Processed food	Reverse percentage yield	Conversion factor^b^	Number of samples	References
Polishing	Whole grains	Polished cereal	0.71	Polished cereal	1.4 ± 0.37	1	5	(EFSA et al. 2019; Scholz et al. 2022)
		Husk	0.29	—	3.4 ± 0.40	—	5	(EFSA et al. 2019; Scholz et al. 2022)
Milling	Whole grains	Bran	0.18	Bran	5.7 ± 0.18	1	3	(Bergström 1994; Bognár 2002; FAO 2000)
		Germ	0.04	Germ	27 ± 11.1	1	3	(EFSA et al. 2019; FAO 2000)
		Endosperm	0.88	Endosperm	1.1 ± 0.29	1	3	(Bognár 2002; EFSA et al. 2019; FAO 2000; Scholz et al. 2022)
Juicing	Citrus fruits	Juice	0.42	Citrus fruit juice	2.4 ± 0.35	1	6	(Bognár 2002; EFSA et al. 2019; FAO 2000; Scholz et al. 2022)
		Pulp	0.58	—	1.7 ± 0.24	—	6	(Bognár 2002; EFSA et al. 2019; FAO 2000; Scholz et al. 2022)
	Pome fruits	Juice	0.42	Pome fruit juice	2.4 ± 1.75	1	4	(Bognár 2002; EFSA et al. 2019; FAO 2000; Scholz et al. 2022)
		Pulp	0.58	—	1.7 ± 0.48	—	4	(Bognár 2002; EFSA et al. 2019; FAO 2000; Scholz et al. 2022)
	Berries and small fruits	Juice	0.63	Berry and small fruit juice	1.6 ± 0.19	1	10	(Bognár 2002; EFSA et al. 2019; FAO 2000; Scholz et al. 2022)
		Pulp	0.38	—	2.7 ± 0.24	—	10	(Bognár 2002; EFSA et al. 2019; FAO 2000; Scholz et al. 2022)
	Stone fruits	Juice	0.63	Stone fruit juice	1.6 ± 0.27	1	5	(Bognár 2002; EFSA et al. 2019; FAO 2000; Scholz et al. 2022)
		Pulp	0.38	—	2.7 ± 0.59	—	5	(Bognár 2002; EFSA et al. 2019; FAO 2000; Scholz et al. 2022)
	Miscellaneous tropical/sub‐tropical fruits	Juice	0.32	Miscellaneous tropical/sub‐tropical fruit juice	3.1 ± 1.73	1	5	(Bognár 2002; EFSA et al. 2019; FAO 2000; Scholz et al. 2022)
		Pulp	0.68	—	1.5 ± 1.03	—	5	(Bognár 2002; EFSA et al. 2019; FAO 2000; Scholz et al. 2022)
	Vegetables	Juice	0.56	Vegetable juice	1.8 ± 0.31	1	9	(Bognár 2002; EFSA et al. 2019; FAO 2000; Scholz et al. 2022)
		Pulp	0.44	—	2.3 ± 0.79	—	9	(Bognár 2002; EFSA et al. 2019; FAO 2000; Scholz et al. 2022)
Vegetable oil production via mechanical pressing	Cotton seeds	Oil	0.12	Cotton seed oil	8.6 ± 1.43	1	5	(FAO 2000; Latif et al. 2007; Orhevba and Efomah 2012; Rojo‐Gutíerrez et al. 2020; Taha and Hassanein 2007)
		Oil cake	0.88	—	1.1 ± 0.42	—	5	(FAO 2000; Latif et al. 2007; Orhevba and Efomah 2012; Rojo‐Gutíerrez et al. 2020; Taha and Hassanein 2007)
	Olives	Oil	0.17	Olive oil	6.1 ± 1.72	1	3	(Abenoza et al. 2013; Inarejos‐García et al. 2009; Sharma et al. 2007)
		Oil cake	0.83	—	1.2 ± 0.67	—	3	(Abenoza et al. 2013; Inarejos‐García et al. 2009; Sharma et al. 2007)
	Peanuts	Oil	0.18	Peanut oil	5.6 ± 0.41	1	2	(Idrus et al. 2017)
		Oil cake	0.82	—	1.2 ± 0.23	—	2	(Idrus et al. 2017)
	Perilla seeds	Oil	0.34	Perilla oil	2.9 ± 0.15	1	5	(Jung et al. 2012; Lee et al. 2023; Oh et al. 2018; Park et al. 2021)
		Oil cake	0.66	—	1.5 ± 0.08	—	5	(Jung et al. 2012; Lee et al. 2023; Oh et al. 2018; Park et al. 2021)
	Rapeseeds	Oil	0.15	Rapeseed oil	6.7	1	1	(Azadmard‐Damirchi et al. 2010)
		Oil cake	0.85	—	1.2	—	1	(Azadmard‐Damirchi et al. 2010)
	Rice bran	Oil	0.04	Rice bran oil	27 ± 5.91	1	2	(Srikaeo and Pradit 2011; Thanonkaew et al. 2012)
		Oil cake	0.96	—	1.0 ± 1.28	—	2	(Srikaeo and Pradit 2011; Thanonkaew et al. 2012)
	Sesame seeds	Oil	0.39	Sesame seed oil	2.6 ± 0.10	1	3	(Park et al. 2021; Ribeiro et al. 2016)
		Oil cake	0.61	—	1.6 ± 0.41	—	3	(Park et al. 2021; Ribeiro et al. 2016)
Vegetable oil production via solvent extraction	Cotton seeds	Oil	0.36	Cotton seed oil	2.8 ± 0.68	1	2	(Latif et al. 2007; Rojo‐Gutíerrez et al. 2020)
		Oil cake	0.64	—	1.6 ± 0.47	—	2	(Latif et al. 2007; Rojo‐Gutíerrez et al. 2020)
	Flaxseeds	Oil	0.26	Flaxseed oil	3.8 ± 0.12	1	2	(FAO 2000; Zanqui et al. 2015)
		Oil cake	0.74	—	1.4 ± 0.31	—	2	(FAO 2000; Zanqui et al. 2015)
	Olives	Oil	0.22	Olive oil	4.5 ± 0.76	1	2	(Akretche‐Kelfat et al. 2017; Bertouche et al. 2013; FAO 2000)
		Oil cake	0.78	—	1.3 ± 0.74	—	2	(Akretche‐Kelfat et al. 2017; Bertouche et al. 2013; FAO 2000)

	Peanuts	Oil	0.43	Peanut oil	2.3 ± 0.14	1	3	(Akretche‐Kelfat et al. 2017; Bertouche et al. 2013; FAO 2000)
		Oil cake	0.57	—	1.7 ± 0.27	—	3	(Akretche‐Kelfat et al. 2017; Bertouche et al. 2013; FAO 2000)
	Perilla seeds	Oil	0.40	Perilla oil	2.5 ± 0.40	1	2	(Jung et al. 2012; Lee et al. 2006)
		Oil cake	0.60	—	1.7 ± 0.23	—	2	(Jung et al. 2012; Lee et al. 2006)
	Rapeseeds	Oil	0.39	Rapeseed oil	2.6 ± 0.03	1	2	(Azadmard‐Damirchi et al. 2010; Guderjan et al. 2007)
		Oil cake	0.61	—	1.6 ± 0.02	—	2	(Azadmard‐Damirchi et al. 2010; Guderjan et al. 2007)
	Rice bran	Oil	0.07	Rice bran oil	15 ± 3.30	1	4	(Amarasinghe and Gangodavilage 2004; Chia et al. 2015; Pourali et al. 2009; Soares et al. 2018)
		Oil cake	0.93	—	1.1 ± 0.99	—	4	(Amarasinghe and Gangodavilage 2004; Chia et al. 2015; Pourali et al. 2009; Soares et al. 2018)
	Sesame seeds	Oil	0.45	Sesame seed oil	2.2 ± 0.16	1	4	(Dim 2013; Ribeiro et al. 2016; Tir et al. 2012; Zahran et al. 2020)
		Oil cake	0.55	—	1.8 ± 0.17	—	4	(Dim 2013; Ribeiro et al. 2016; Tir et al. 2012; Zahran et al. 2020)
	Soybeans	Oil	0.19	Soybean oil	5.3 ± 0.20	1	6	(Bertouche et al. 2013; Claux et al. 2021; FAO 2000; Nikolic et al. 2009; Nodar et al. 2002; Potrich et al. 2020)
Oil cake		0.81	—	1.2 ± 0.23	—	6	(Bertouche et al. 2013; Claux et al. 2021; FAO 2000; Nikolic et al. 2009; Nodar et al. 2002; Potrich et al. 2020)
Butter production	Milk	Skim milk	0.90	Skim milk	1.1 ± 0.22	1	2	(FAO 2000; Milk 2014)
				Skim milk powder	11	9.7	1	(FAO 2000)
		Cream	0.10	Cream	10 ± 2.11	1	3	(Bergamaschi et al. 2016; FAO 2000; Milk 2014)
				Butter	22 ± 5.73	2.1	4	(FAO 2000; Faye and Konuspayeva 2012; Gebremedhin et al. 2014; Oeffner et al. 2013)
Cheese production	Milk	Curd	0.10	Camembert cheese	10 ± 2.83	1.0	3	(Durham et al. 2015; Galli et al. 2019; Zikiou and Zidoune 2019)
				Cheddar cheese	11 ± 0.43	1.1	2	(Auldist et al. 2016; Avramis et al. 2003)
				Cottage cheese	5.6 ± 1.51	0.6	5	(Hallab et al. 2007; Mahami et al. 2012; Makhal et al. 2013; Rasheed et al. 2016; Sharma and Vaidya 2018)
				Cream cheese	3.8 ± 1.52	0.4	3	(Bahrami et al. 2015; Han et al. 2008b; Jeon et al. 2012)
				Gouda cheese	10 ± 0.80	1.0	4	(Domagala et al. 2022; Durham et al. 2015; Llorente et al. 2014; Sulieman et al. 2018)
				Mozzarella cheese (fresh and processed)	9.1 ± 2.03	0.9	12	(Durham et al. 2015; El Owni and Osman 2009; Franceschi et al. 2020; Francolino et al. 2010; Govindasamy‐Lucey et al. 2007; Govindasamy‐Lucey et al. 2006; Gulati et al. 2018; Lilbaek et al. 2006; Luiz et al. 2022; Metzger et al. 2000; Oeffner et al. 2013)
		Whey	0.90	Whey protein powder	18 ± 1.81	16	3	(Bédas et al. 2017; EFSA et al. 2019; Hurst et al. 1990; Riera et al. 2016)
Powdered egg product production	Eggs	Egg white	0.67	Egg white	1.5	1	1	(USDA 1992)
				Egg white powder	13	8.9	1	(USDA 1992)
		Egg yolk	0.33	Egg yolk	3.0 ± 0.11	1	2	(USDA 1992; Wei et al. 2019)
				Egg yolk powder	5.9 ± 0.36	1.9	2	(USDA 1992; Wei et al. 2019)
Soy sauce/paste production	Soybeans	Soy sauce	0.28	Soy sauce	0.3 ± 0.03	0.1	4	(Choi et al. 2000; FAO 2000; Jeil Bio Tech 2013; Shin et al. 2014)
		Soybean paste	0.72	Soybean paste	0.4 ± 0.24	0.3	2	(Jeil Bio Tech 2013; Kim et al. 2017)
Soybean curd production	Soybeans	Soy pulp	0.38	Soy pulp	0.4	0.2	1	(Han et al. 2020)
		Soy milk	0.62	Soy milk	0.2 ± 0.05	0.1	2	(Jackson et al. 2002; Sung et al. 2022)
				Bean curd	0.4 ± 0.09	0.2	10	(Han et al. 2020; Jackson et al. 2002; Kim et al. 2017, 2010; Kim and Lee 2007; Lee et al. 2011a; Mujoo et al. 2002, 2003; Sim et al. 2020; Yoo 2011)
Starch production	Cereals	Starch slurry	0.86	Cereal starch	1.5 ± 0.18	1.3	6	(EFSA et al. 2019; FAO 2000; Knight and Olson 1984; Scholz et al. 2022)
		Protein residue	0.14	Protein isolate	3.0 ± 0.94	0.4	6	(EFSA et al. 2019; FAO 2000; Knight and Olson 1984; Scholz et al. 2022)
	Starchy roots/tubers	Starch slurry	0.58	Starchy root/tuber starch	5.2 ± 1.33	3.0	4	(Chavalparit and Ongwandee 2009; FAO 2000; Rahman et al. 2003; Scholz et al. 2022)
		Protein residue	0.42	Protein isolate	1.2 ± 0.98	0.5	4	(Chavalparit and Ongwandee 2009; FAO 2000; Rahman et al. 2003; Scholz et al. 2022)

*Note:* Conversion factors are presented as the mean ± standard deviation.

^a^ The partition ratio refers to the ratio of partitioning multiple segments as a result of initial separation from starting raw materials.

^b^ Conversion factors were calculated by multiplying partition ratios with reverse percentage yields (weight of fractionated food ingredient relative to that of the finished processed food). Values from previous studies were averaged to generate factors.

The remaining 50 factors were generated using partition ratios and percentage yields, as targeted processed foods are produced after being partitioned from their initial food ingredients (Section 2.4.1). Processed foods in this category can be subdivided according to post‐separation downstream steps. For instance, certain foods, including polished cereals, milled products, egg yolk/egg white, vegetable/fruit juices, and vegetable oils, are solely produced via physical separation. In these cases, a conversion factor of 1 was assigned, as downstream procedures do not tend to elicit significant weight changes until processed into final products (Table 3). Alternatively, as partition ratios become equal to the yield ratios of the final products generated from individual fractions after separation from raw materials, a comparable result can also be obtained by multiplying partition ratios (yield ratios) by reverse percentage yields (Equation (2)). Partition and yield ratios were also tabulated in situations where partition ratio data were evidently useful, as described in the Discussion.

The partition ratios for juice and oil, relative to their raw materials, were affected by the raw materials' physical and chemical properties. Those for citruses, pomes, and tropical fruits were lower (0.32–0.42) than those for vegetables, berries, and stone fruits (0.56–0.63). Overall, oils separated (processed) via mechanical pressing generated lower partition ratios (0.04–0.39) than those subjected to solvent extraction (0.07–0.45) owing to additional oil fractionation from oil cakes during solvent extraction (Table 3). However, perilla and sesame seed pressing produced a relatively large oil fraction (perilla seeds: 0.34, sesame seeds: 0.39), which was slightly smaller than that produced via solvent extraction (perilla seeds: 0.40, sesame seeds: 0.45). Within the same solvent method of oil production, cotton seeds, rapeseeds, perilla seeds, sesame seeds, and peanuts yielded larger oil fractions (0.36–0.45) than other raw materials (approximately 0.2; soybeans, flaxseeds, and olives), reflecting their fat content (NAS 2023; USDA 2022). The oil fraction derived from rice bran was extremely small, that is, merely 7% of the raw materials (rice bran), reflecting its meager fat content and the low oil‐separation efficiency from rice bran (Table 3).

On the other hand, certain processed foods undergo further processing during downstream procedures, such as dehydration and evaporation, as in the cases of egg yolk powder, egg white powder, skim milk powder, butter, cheese, and starch. In cheese products, PRCFs approximated “1” (camembert, cheddar, and gouda cheese) or lower (cream and cottage cheese). Contrastingly, they were high (approximately 2) for butter and egg yolk powder and extremely high (approximately 9) for egg white powder and skim milk powder (Table 3 and Figure 2). Therefore, their conversion factors depended on the extent of water removal and the composition of fractions. In other processed foods (e.g., soy milk, tofu, soy sauce, and soybean paste), processing entails the soaking and hydration of the initial ingredients before separation, thus increasing their initial weights. Consequently, the conversion factors for soybean‐derived products were considerably low (0.1–0.3; Table 3).

### 3.2 PRCFs Generated Using the Migration Rate Method

Certain processed foods, such as alcoholic beverages and vinegars, are manufactured via complicated procedures, rendering conversion based on simple weight changes during processing challenging. In addition, processed foods prepared via brewing or extraction contain a negligible portion of their initial ingredients, as a major part would have been discarded. Therefore, in such circumstances, migration rates were calculated using a specific component traceable from the initial ingredient to the final product. A specific component was selected for each processed food based on its abundance and stability as follows: carbohydrates, α‐acids, total solids, and total polyphenols for alcoholic beverages and vinegars; hop; broths/stocks; and assorted tea types, respectively (Section 2.4.2).

**TABLE 4:** Processed‐to‐raw food conversion factors generated using the migration rates of specific components.

Processing procedure	Processed foods	Initial food ingredients	Migration rate		Number of samples	References
			Specific component	Conversion factor^a^		
Alcoholic beverage production	Fermented alcoholic beverages (Korean rice wine, and wine)	Cereals	Carbohydrate	0.1350 ± 0.013	5	(Cheon et al. 2013; Kim and Eun 2012; NAS 2023; USDA 2022)
		Fruits	Carbohydrate	1.8751 ± 0.512	10	(EFSA et al. 2019; NAS 2023; USDA 2022)
	Fermented alcoholic beverages (beer)	Cereals	Carbohydrate	0.1259 ± 0.003	2	(NAS 2023; USDA 2022)
		Hop	α‐acid	0.0021 ± 0.001	20	(Bailey et al. 2009; Bober et al. 2020; Jaskula et al. 2007; Kemp et al. 2021; Klimczak and Cioch‐Skoneczny 2023; Machado et al. 2019; Oladokun et al. 2017; Oladokun et al. 2016)
	Distilled alcoholic beverages (vodka, whiskey, distilled soju, and brandy)	Cereals and starchy roots/tubers	Carbohydrate	1.3823 ± 0.820	13	(Kwon et al. 2023a, 2023b; Lee et al. 2017a, 2014, 2017b; Moon and Cheong 2018; NAS 2023; USDA 2022)
		Fruits	Carbohydrate	5.8652 ± 1.464	8	(EFSA et al. 2019; FAO 2000; Jackson 2009; NAS 2023; USDA 2022)
	Distilled and diluted alcoholic beverages (diluted soju, diluted vodka, and light rum)	Cereals and starchy roots/tubers	Carbohydrate	0.8686 ± 0.547	7	(NAS 2023; USDA 2022)
Vinegar production	Vinegars	Cereals	Carbohydrate	0.3228 ± 0.184	7	(Joo et al. 2009; Lee et al. 2011b; Na et al. 2013; NAS 2023; Yoon et al. 2010)
		Fruits	Carbohydrate	0.6920 ± 0.252	6	(EFSA et al. 2019; Kang 2006; Na et al. 2013; NAS 2023)
Broth/stock production	Broths/stocks	Beef bones	Total solid	0.0156 ± 0.007	9	(Kim et al. 2014a; Moon et al. 2015; Yoon et al. 2015)
		Poultry meat and offal	Total solid	0.0197 ± 0.012	13	(Choi 2011, 2012; Kim et al. 2023, 2008, 2011; Lee et al. 2003)
		Mixed meat and offal	Total solid	0.0514 ± 0.037	5	(Choi et al. 2008b; Choi and Kim 2010; Kim et al. 2004; Kim et al. 2013a; Shin and Lee 2011)
		Anchovies (dried)	Total solid	0.0049 ± 0.004	3	(Kim et al. 2013a; Kim and Park 2013; Lee and Ryu 2014; Shin and Lee 2011)
		Fish other than anchovies (dried)	Total solid	0.0269 ± 0.024	9	(Bae et al. 2007; Jun et al. 2020, 2021; Kang et al. 2009; Kim et al. 2013b, 2014b, 2012)
		Seaweed (dried)	Total solid	0.0043 ± 0.002	3	(Jang et al. 2019; Shin and Lee 2011)
		Vegetables	Total solid	0.0139 ± 0.007	4	(Han et al. 2008c)

^a^ Conversion factors were calculated using the migration rates of specific components. For alcoholic beverages, the migration rates of carbohydrates (alcohol) were calculated as the relative ratio of alcohol per volume of produced alcoholic beverage to the carbohydrate weight of the initial ingredient multiplied by 0.51 (theoretical fermentation yield). For vinegars, the migration rates of carbohydrates (acetic acid) were calculated as the relative ratio of acetic acid per volume of produced vinegar to the carbohydrate weight of the initial food ingredient multiplied by 0.67 (theoretical fermentation yield). For broth/stock, the migration rates of total solids were calculated as the total solid weight per 100 g of produced food relative to the total solid weight of the initial food ingredient.

**TABLE 5:** Processed‐to‐raw food conversion factors generated using the migration rates of total polyphenols measured from various tea materials and their infusions.

Processing procedure	Plant part used to prepare teas/infusions	Initial food ingredient	Conversion factor^a^	Total polyphenol content (mg GAE/g dry weight) in tea raw material^b^				
				Current study		Previous study		
				Range	Number of samples	Range	Number of samples	References
Tea brewing	Leaf	Black tea	0.0076 ± 0.0013	94.5–105 (98.1)	3	29.3–135 (84.7)	8	(AlHafez et al. 2014; Chan et al. 2010; Jeong et al. 2009; Khokhar and Magnusdottir 2002; Oh et al. 2013)
		Green tea	0.0119 ± 0.0003	90.5–112 (99.2)	3	65.8–183 (123)	8	(Cai et al. 2004; Chan et al. 2010; Horzic et al. 2009; Jeong et al. 2009; Khokhar and Magnusdottir 2002; Oh et al. 2013)
		Peppermint	0.0045 ± 0.0010	27.1–62.7 (49.4)	3	13.2–231 (49.5)	55	(Chan et al. 2010; Dogan et al. 2010; Dorman et al. 2003; Farnad et al. 2014; Fecka and Turek 2007; Kirca and Arslan 2008; Kratchanova et al. 2010; Liu et al. 2008; Oh et al. 2013; Petkova et al. 2017; Sarikurkcu et al. 2017)
		Rooibos	0.0100 ± 0.0001	43.9, 57.8 (50.8)	2	16.7–271 (66.4)	10	(Chan et al. 2010; Epure et al. 2019; Joubert and de Beer 2012; Oh et al. 2013; Santos et al. 2016)
	Flower	Chamomile	0.0046 ± 0.0015	39.8, 48.0 (43.9)	2	2.08–46.7 (17.2)	6	(Ayhan 2021; Chan et al. 2010; Kratchanova et al. 2010; Prakash et al. 2007; Roby et al. 2013; Seddik et al. 2013)
		Hibiscus	0.0043 ± 0.0001	31.3, 34.7 (33.0)	2	15.9–100 (56.8)	32	(Borrás‐Linares et al. 2015; Lee et al. 2008b; Patel et al. 2023; Singh et al. 2021; Surveswaran et al. 2007)

	Root	Burdock (roasted)	0.0019 ± 0.0011	14.2, 16.0 (15.1)	2	21.4–32.4 (27.5)	4	(Chociej et al. 2024; Ferracane et al. 2010; Kratchanova et al. 2010; Petkova et al. 2017)
		Solomon's seal (roasted)	0.0036 ± 0.0003	10.9, 13.4 (12.2)	2	11.1–31.7 (18.6)	3	(Ahongshangbam et al. 2014; Li et al. 2018; Liu et al. 2008)
	Fruit/seed	Barley (roasted)	0.0024 ± 0.0010	10.2–10.9 (10.4)	3	1.30–19.9 (5.68)	44	(Hodzic et al. 2009; Holtekjolen et al. 2006; Mansouri et al. 2021; Patel et al. 2023; Yoshida et al. 2010)
		Cassia seed (roasted)	0.0016 ± 0.0009	19.4–21.7 (20.8)	3	6.40–22.6 (13.2)	5	(Cai et al. 2004; Lee et al. 2008b; Liu et al. 2008; Patel et al. 2023; Surveswaran et al. 2007)
		Rosehip	0.0033 ± 0.0007	58.4, 69.7 (64.0)	2	5.09–96.0 (42.8)	22	(Angelov et al. 2014; Ercisli 2007; Ersoy et al. 2015; Fascella et al. 2019; Gao et al. 2000; Petkova et al. 2017; Soare et al. 2015; Su et al. 2007)
Bark	Cinnamon	0.0005 ± 0.0001	30.0–51.4 (39.1)	3	18.6–245 (89.6)	11	(Abeysekera et al. 2013; Assefa et al. 2018; Cai et al. 2004; Chimbetete et al. 2019; Lu et al. 2011; Prakash et al. 2007; Rayess et al. 2023; Saranya et al. 2017; Shan et al. 2005; Słowianek and Leszczyńska 2016; Su et al. 2007)

^a^ Conversion factors were calculated as an average of the total polyphenol migration rates (relative ratio of the total polyphenol weight per volume of brewed tea to the total polyphenol weight of the initial tea material) of two to three samples in each initial ingredient (Table 1). Values are presented as the mean ± standard deviation.

^b^ The total polyphenol content is expressed in mg gallic acid equivalent (GAE)/g dry weight in a range with the average indicated in parentheses.

In total, 38 PRCFs were generated using the migration rate method (Tables 4, 5, 6). In alcoholic beverages, distilled types exhibited higher conversion factors (cereals: 1.38, fruits: 5.86) for the same initial food ingredient than their fermented types (cereals: 0.14, fruits: 1.88), as shown in Table 4. In addition, regardless of the alcohol‐manufacturing process, cereal‐derived alcoholic beverages yielded lower conversion factors (fermented: 0.14, distilled: 1.38) than fruit‐derived ones (fermented: 1.88, distilled: 5.86). Distilled and diluted beverages generated a lower value (cereals: 0.87) than distilled alcoholic beverages (cereals; 1.38) for the same raw material owing to water addition. In vinegars, cereal‐based products yielded a lower conversion factor (0.32) than fruit‐based ones (0.69), consistent with alcoholic beverages.

**TABLE 6:** Processed‐to‐raw food conversion factors generated using the migration rates of total polyphenols directly measured from various spices and their prepared stocks.

Processing procedure	Plant parts used to prepare stocks	Initial food ingredient	Conversion factor^a^	Total polyphenol content (mg GAE/g dry weight) in spice^b^				
				Current study		Previous study		
				Mean	Number of samples	Range	Number of samples	References
	Leaf	Bay leaf	0.0036	35.3	1	30.9–92.0 (49.7)	8	(Albayrak et al. 2012; Assefa et al. 2018; Hinneburg et al. 2006; Kirca and Arslan 2008; Kratchanova et al. 2010; Lu et al. 2011; Shan et al. 2005; Słowianek and Leszczyńska 2016)
	Flower bud	Clove	0.0078	131	1	19.1–265 (165)	9	(Adaramola and Onigbinde 2016; Assefa et al. 2018; Chimbetete et al. 2019; Gülçin et al. 2004; Liu et al. 2008; Przygodzka et al. 2014; Rayess et al. 2023; Słowianek and Leszczyńska 2016; Temesgen et al. 2022)
	Fruit/seed (hard)	Black pepper	0.0007	17.4	1	1.32–38.2 (10.8)	12	(Assefa et al. 2018; Feng et al. 2020; Lee et al. 2020; Liu et al. 2008; Lu et al. 2011; Patel et al. 2023; Rayess et al. 2023; Saranya et al. 2017; Shan et al. 2005; Słowianek and Leszczyńska 2016; Su et al. 2007; Surveswaran et al. 2007)
		Coriander	0.0122	5.52	1	0.94–12.1 (4.64)	21	(Agrawal et al. 2016; Assefa et al. 2018; Deepa et al. 2013; Ezez and Kussie 2022; Hameed et al. 2019; Kirca and Arslan 2008; Msaada et al. 2017; Ninfali et al. 2005; Patel et al. 2023; Rayess et al. 2023; Sriti et al. 2024; Surveswaran et al. 2007; Tibebe et al. 2024; Wangensteen et al. 2004)
		Nutmeg	0.0065	12.5	1	2.62–37.3 (15.3)	11	(Assefa et al. 2018; Liu et al. 2008; Lu et al. 2011; Prakash et al. 2007; Przygodzka et al. 2014; Rayess et al. 2023; Shan et al. 2005; Słowianek and Leszczyńska 2016; Su et al. 2007; Surveswaran et al. 2007; Tan et al. 2013)
		Star anise	0.0021	27.1	1	13.2–53.9 (24.6)	7	(Albayrak et al. 2012; Assefa et al. 2018; Liu et al. 2008; Lu et al. 2011; Patel et al. 2023; Shan et al. 2005; Surveswaran et al. 2007)

	Fruit/seed (soft)	Caraway	0.0043	8.40	1	3.27–37.4 (12.0)	5	(Assefa et al. 2018; Hinneburg et al. 2006; Rayess et al. 2023; Shan et al. 2005; Słowianek and Leszczyńska 2016)
		Cumin	0.0033	14.1	1	2.30–9.00 (6.82)	8	(Assefa et al. 2018; Lu et al. 2011; Ninfali et al. 2005; Proestos et al. 2006; Rayess et al. 2023; Shan et al. 2005; Surveswaran et al. 2007; Thippeswamy and Naidu 2005)
		Fennel	0.0071	12.9	1	3.40–51.1 (21.4)	53	(Albayrak et al. 2012; Assefa et al. 2018; Ben Abdesslem et al. 2021; Cai et al. 2004; Faudale et al. 2008; Hinneburg et al. 2006; Khammassi et al. 2022; Liu et al. 2008; Lu et al. 2011; Patel et al. 2023; Roby et al. 2013; Surveswaran et al. 2007; Yaldiz and Camlica 2019)
Bark	Cinnamon	0.0019 ± 0.0004	39.6	3	18.6–245 (89.6)	11	(Abeysekera et al. 2013; Assefa et al. 2018; Cai et al. 2004; Chimbetete et al. 2019; Lu et al. 2011; Prakash et al. 2007; Rayess et al. 2023; Saranya et al. 2017; Shan et al. 2005; Słowianek and Leszczyńska 2016; Su et al. 2007)

^a^ Conversion factors were calculated as an average of triplicate total polyphenol migration rates (relative ratio of the total polyphenol weight per volume of spice stock to the total polyphenol weight of the initial spice material). Values are presented as the mean ± standard deviation.

^b^ The total polyphenol content is expressed in mg gallic acid equivalent (GAE)/g dry weight as a mean (current study) or in a range with the average of total polyphenol contents in parentheses (previous study).

The PRCFs for food products prepared via extraction were extremely low (< 0.1; Tables 4, 5, 6 and Figure 2). Within this processed food category, the PRCFs for broths/stocks prepared from fresh meats and vegetables (0.0156–0.0514) exceeded those for hop extracted in beer (0.0021), tea infusions (≤ 0.01), and broths/stocks prepared from anchovy, seaweed, or spices (≤ 0.01; Tables 4, 5, 6). PRCFs also varied according to the plant part used to prepare tea infusions. They were relatively high for teas brewed from leaves (0.0045–0.0119), followed by those for teas brewed from flowers (0.0043–0.0046) compared with those for teas infused from roots (0.0019–0.0036), fruits/seeds (0.0016–0.0033), or bark (0.0005). In comparison, the effects of raw material parts were not noticeable in PRCFs derived for stocks prepared using diverse spices. Stock preparation requires a considerably longer extraction time than tea making; thus, the physicochemical nature of raw materials may affect extraction efficiency less. Cinnamon was used to prepare both teas and stocks, and stocks (0.0019) requiring a longer preparation time yielded a higher PRCF than teas (0.0005). Regarding initial ingredients, the polyphenol composition established in the current study is comparable to that obtained in previous studies (Tables 5 and 6).

## 4 Discussion

In the current study, PRCFs were generated to adequately convert the weights of processed foods (actually consumed amounts) to those of their constituent raw food materials (estimated amounts). Moreover, conversion factors were generated to cover various processed foods produced via diverse procedures, ranging from grinding to household cooking procedures and to more sophisticated industrial processing techniques. To generate PRCFs, two methods were primarily used: (1) the percentage yield method, wherein the weight ratio of initial raw ingredients to final processed products was calculated, and (2) the migration rate method, which involved calculating the content ratio of specific components in final processed foods relative to their initial raw ingredients (Figure 1).

For most food processing procedures, PRCFs were derived using the percentage yield or modified percentage yield method. The modified percentage yield method considered partition ratios in addition to percentage yields, as numerous processed foods are produced after undergoing separation from initial raw materials. This modification minimizes overestimation by summing all individual compartments as weights comparable to those of their whole ingredients, thus more accurately converting to their initial weights, compared with the method that exclusively uses percentage yield. Moreover, partition ratios can be replaced or modified with the migration ratios of chemicals, facilitating a more accurate estimation of dietary exposure to target chemicals. For example, cholesterol is exclusively present in egg yolk (NAS 2023; USDA 2022); that is, in eggs, the cholesterol partition ratio is “1” for egg yolk and “0” for egg white. Therefore, the amount of egg yolk is equivalent to that of the whole egg in terms of cholesterol level. Similarly, if certain hazardous materials are present in one compartment exclusively (or primarily) or migrate during processing to one compartment relative to others, partition ratios can subsequently be replaced with migration ratios of “1” for the compartment where chemicals are present (or migrate) and “0” for the remaining compartments. Therefore, if the migration (or distribution) ratios of food chemicals for individual compartments relative to those for the entire food are known, they can be used to replace partition ratios to accommodate a broad range of target chemical properties, further enabling the accurate assessment of their dietary exposure.

Overall, water was a major contributor to PRCF changes (Figure 2), exhibiting consistency with previous studies (Adikari and Thamilini 2018; Kweon 2012). Dehydration, evaporation/condensation, roasting/sautéing, and canning reduce the weight of the product compared with that of the starting materials, albeit by varying degrees (Tables 2 and 3 and Figure 2). Notably, the PRCFs for processed foods prepared via dehydration exhibited substantially high values (range: 2.2–18; Table 2 and Figure 2). Dehydration requires prolonged heating, subsequently causing greater moisture loss during processing compared with other heating methods. Nonetheless, dehydration rates vary with food type. This variation can be explained by the different water contents and compositions across foods as well as the size and shape of the initial ingredient, thus affecting the water‐holding capacity of raw materials. For example, meat contains abundant proteins. Certain amino acids (e.g., asparagine and glutamic acid) can bind water (Kudryashov and Kudryashova 2023). As such, myosin, which is rich in these amino acids, possesses a high water‐holding capacity (Zayas 1997). Water molecules that are bound and held by the polar groups of proteins constitute tightly bound water, thus increasing the water‐holding capacity of meats (Kudryashov and Kudryashova 2023). Conversely, fruiting vegetables (e.g., tomatoes and bell pepper) and mushrooms contain abundant water, contributing to greater post‐dehydration weight changes.

Conversely, hydration or water addition elicits an increase in the weight of the final product relative to that of the starting materials, reducing the conversion factors (Tables 2 and 3 and Figure 2). For example, during soy paste and soy sauce production, a vast quantity of water is added to soybean at the beginning of the production process, leading to low conversion factors for the final products (Choi et al. 2000; Kim et al. 2017). Therefore, a lesser amount of soybean is actually consumed than that of soy sauce or soybean paste ingested. During boiling, water can be absorbed or squeezed out depending on the food type. For example, in a previous study, food weight approximately doubled during the boiling of low‐water‐content cereals and legumes owing to water absorption (Bognár 2002). Conversely, the boiling of vegetables, fruits, meats, and fish causes the collapse of cells and subsequent release of water from foods into the surrounding water (Andersson et al. 2022), resulting in elevated conversion factors (Table 2).

Partition ratios are potentially important when estimating dietary chemical exposure from processed foods produced from an individual compartment of the initial food raw materials, although a conversion factor of 1 was uniformly assigned to processed foods produced via physical separation. The partition ratios for oil and starch were effectively reflected by their content in food raw materials (Table 3). Moreover, the physicochemical properties of the food types determined the fraction ratios of oil and starch. For example, the softening of rice bran by mild heating increased the oil fraction percentage from approximately 4% to 7% (Srikaeo and Pradit 2011). In addition, the interactions of oil or starch with other components in raw materials may potentially influence the fraction ratio of the oil or starch phase. Overall, the fractionation of oil‐bearing crops predominantly using physical forces yielded a lower liquid‐phase (oil part) ratio than solvent extraction, which entails additional fractionation from the solid phase (oil cake) (Table 3). Likewise, separation of the liquid–solid phase from fruits or vegetables resulted in a sizeable remainder of pulp (Table 3), and it was immensely affected by the water and fiber content of their raw materials (Prestes et al. 2023).

The migration rate method was applied when weight change could not easily be traced during processing, as in the case of alcoholic beverage and vinegar manufacture as well as extract preparation from various raw materials. Under these circumstances, specific components were tracked instead based on the assumption that they would represent the entire food. Alcohol or acetic acid enrichment during alcohol or vinegar production increased PRCF levels (Table 4 and Figure 2). In alcoholic beverages, conversion factors were lower when cereals were used as starting materials than when fruits were employed under the same alcohol preparation conditions (fermented or distilled). Similarly, cereal‐derived vinegars exhibited lower conversion factors than fruit‐derived ones (Table 4). For both alcoholic beverages and vinegars, carbohydrate (alcohol/acetic acid) was selected as a traceable component from initial ingredients to final products. The wheat, rice, and barley used as raw materials for alcoholic beverage and vinegar production comprise 70%–80% carbohydrate compared with the 30%–60% in fruits (e.g., grapes and apples) and starchy root vegetables (e.g., sweet potato) (McCleary and McLoughlin 2021; NAS 2023; USDA 2022). Therefore, the larger percentage of carbohydrates in cereals augments alcohol and vinegar production compared with that in fruits.

Conversely, multiple extracts prepared by extracting diverse food ingredients exhibited extremely low PRCF values. However, extraction conditions (e.g., time and temperature) and food type influence extraction efficiency and subsequently PRCFs. Overall, a longer time and higher temperature enhance extraction efficiency, yielding higher PRCFs for broths/stocks prepared from fresh meats and vegetables over a prolonged period (typically ≥ 2 h) than those of tea infusions prepared from dried matter within minutes at lukewarm or fairly high temperatures (Tables 4 and 5). Anchovy and seaweed broths are typically prepared from dried materials over a comparatively brief period (< 1/2 h), yielding lower PRCFs than broths/stocks prepared using fresh meats or vegetables (Kim et al. 2013a). Ingredient type also affects the migration rate when preparing teas and herbal infusions because the size, shape, and structural differences of different parts may affect extraction efficiency.

Notwithstanding, the current study has several limitations associated with the generation of PRCFs using both the percentage yield (relative weights) and migration rate (relative content of a specific component) methods. During processing, weight changes are exceedingly influenced by preparation conditions, such as time, temperature, and food type (Mujoo et al. 2003; Nikolic et al. 2009; Sharma et al. 2007; Thanonkaew et al. 2012). In the current study, conversion factors were generated separately for subsets of processed foods refined by factors affecting percentage yield (e.g., the processing technique utilized and food type) to reflect variations in the major processing conditions and physicochemical properties of various food raw materials. In addition, conversion factors were adopted using the mean values of different study results, which may help reflect the actual variation in percentage yield and facilitate a more realistic estimation. However, processing conditions and composition presumably vary within the same processing method across similar food ingredient types, contributing to the over‐ or underestimation of actual initial weights.

Likewise, processing conditions influence the migration rates of food components (Machado et al. 2019; Santos et al. 2016). Furthermore, species and cultivating or rearing conditions potentially affect the contents of selected components in the initial raw ingredient (Park et al. 2024; Pixley and Bjarnason 2002; Simkova et al. 2013). Additionally, in this method, the selection of a traceable component is critical for accurate estimation. Therefore, components were selected based on their abundance in both state and stability. Nevertheless, no component can entirely represent the food ingredient itself, although the migration rate method may better reproduce real consumption quantities when direct measurement is inapplicable. Overall, conversion factors derived from the migration rate method are potentially prone to more errors than those generated from percentage yields when estimating initial weights. Therefore, prioritizing the direct analysis of target chemicals from processed foods in this category (e.g., vinegars, alcoholic beverages, and extracts) may be preferable to making an indirect estimation from food intake, if analytical methods are available.

In summary, the current study generated a total of 120 PRCFs, which were influenced by processing procedures and food types, to convert the weight of processed food into that of raw food (Figure 2), thus adequately estimating the amount of food ingredients from the quantity of consumed processed foods. Moreover, considering partition ratios enables us to adjust for food chemicals with various physicochemical properties, thus imparting versatility to conversion factors. The conversion factors generated in the current study will serve as a valuable tool for accurately estimating food intake, thereby facilitating adequate dietary exposure assessment associated with diverse food chemicals, including pesticide residues, food contaminants, and nutrients.

## Author Contributions

**Jiyun Baek:** data curation (lead), formal analysis (lead). **Yerim Han:** data curation (supporting). **Chaehyun Kim:** data curation (supporting). **You Rim Kang:** data curation (supporting). **Seung Hui Baik:** data curation (supporting). **Yoon Jung Park:** supervision (supporting). **Ji‐Myung Kim:** supervision (supporting), writing – review and editing (supporting). **Youngjoo Kwon:** conceptualization (lead), funding acquisition (lead), supervision (lead), writing – original draft (lead), writing – review and editing (lead).

## Conflicts of Interest

The authors declare no conflicts of interest.

## Data Availability

The data that support the findings of this study are available on request from the corresponding author.
